# Remote Symptom Monitoring to Enhance the Delivery of Palliative Cancer Care in Low-Resource Settings: Emerging Approaches from Africa

**DOI:** 10.3390/ijerph20247190

**Published:** 2023-12-17

**Authors:** Omolola Salako, Adaorah Enyi, Susan Miesfeldt, Johnblack K. Kabukye, Mamsau Ngoma, Eve Namisango, Virginia LeBaron, Chenjerai Sisimayi, Bassey Ebenso, Karl A. Lorenz, Yan Wang, Julie Ryan Wolf, Corina van den Hurk, Matthew Allsop

**Affiliations:** 1 Radiation Biology, Radiotherapy and Radiodiagnosis (RBRR) Digital Health Hub, College of Medicine, Lagos University Teaching Hospital, Lagos 102215, Nigeria; lolasalako@unilag.edu.ng; 2 Oncopadi Technologies, Lagos 105102, Nigeria; dradaorah@oncopadi.com; 3 Medical Oncology, Maine Medical Center, MaineHealth Cancer Care Center, Scarborough, ME 04106, USA; susan.miesfeldt@mainehealth.org; 4 Uganda Cancer Institute, Upper Mulago Hill Road, Kampala P.O. Box 3935, Uganda; jkabukye@gmail.com; 5 Swedish Program for ICT in Developing Regions (SPIDER), Department of Computer and Systems Sciences (DSV), Stockholm University, 164 55 Stockholm, Sweden; 6 Ocean Road Cancer Institute, Dar es Salaam P.O. Box 3592, Tanzania; mamsaungoma@yahoo.com; 7 African Palliative Care Association, Kampala P.O. Box 72518, Uganda; eve.namisango@africanpalliativecare.org; 8 School of Nursing, University of Virginia, Charlottesville, VA 22903, USA; vtl6k@virginia.edu; 9 Department of Mathematics and Applied Mathematics, University of Johannesburg, Johannesburg 2006, South Africa; chenjerai.sisimayi@gmail.com; 10 School of Medicine, University of Leeds, Leeds LS2 9LU, UK; b.e.ebenso@leeds.ac.uk; 11 Ci2i, United States Department of Veterans Affairs, Menlo Park, CA 94025, USA; kalorenz@stanford.edu; 12 Primary Care and Population Health, Stanford School of Medicine, Stanford, CA 94305, USA; 13 School of Nursing, University of Pittsburgh, Pittsburgh, PA 15213, USA; yaw75@pitt.edu; 14 Departments of Dermatology and Radiation Oncology, School of Medicine and Dentistry, University of Rochester Medical Center, Rochester, NY 14642, USA; julie_ryan@urmc.rochester.edu; 15 R&D Department, Netherlands Comprehensive Cancer Organisation, 3501 DB Utrecht, The Netherlands; c.vandenhurk@iknl.nl

**Keywords:** cancer care, Africa, digital health, remote monitoring

## Abstract

This paper brings together researchers, clinicians, technology developers and digital innovators to outline current applications of remote symptom monitoring being developed for palliative cancer care delivery in Africa. We outline three remote symptom monitoring approaches from three countries, highlighting their models of delivery and intended outcomes, and draw on their experiences of implementation to guide further developments and evaluations of this approach for palliative cancer care in the region. Through highlighting these experiences and priority areas for future research, we hope to steer efforts to develop and optimise remote symptom monitoring for palliative cancer care in Africa.

## 1. Introduction

There are critical gaps that have been highlighted in the evidence base regarding the delivery of cancer care in low- and middle-income countries (LMICs) [1]. This includes the need to (i) explore how access to, the affordability of, and outcomes of cancer treatments can be improved, (ii) enhance quality improvement and implementation research, and (iii) explore ways that technology can be used to improve access to and the delivery of cancer care [1,2]. We outline recent developments in remote symptom monitoring and their use in cancer care in sub-Saharan Africa (SSA) that may contribute to the development of evidence across these three areas. 

LMIC settings are facing a growing cancer burden comparable to high-income country settings [3], but there are multiple drivers for a regional focus on SSA; the increasing risk of cancer death in the region, the predominance of late-stage cancer at presentation, and the lack of palliative care to meet population need. In 2020, the cumulative risk of death from cancer among women in Africa was similar to that of regions including Northern America and the highest-income countries of Europe [4]. However, cancer care in SSA is characterised by late-stage clinical presentation, limited funding, and restricted access to curative therapies; up to 80% of cancers are advanced and incurable at the time of diagnosis [5,6,7,8]. Therefore, palliative care is crucial to cancer care in SSA, providing an approach to improving both physical and psychological health-related quality of life (HRQoL), with evidence for survival benefits in high-income settings [9]. However, despite the overwhelming demand, the provision of palliative care in SSA is very limited. Of the 44 countries in SSA assessed in 2017, 27 had some level of palliative care provision (with most reporting very isolated and limited provision), and 17 countries had no provision [10]. When present, palliative care is typically delivered through government-funded inpatient care and outpatient clinics [11] or through donor-funded standalone palliative care organisations [12]. Whilst significant progress has been made in developing palliative care services across SSA [13], less than 10% of people who could benefit from palliation are able to access it [14].

In this article, we use the term palliative cancer care to refer to the integration of palliative care specifically for people living with cancer [15]. Palliative care may have relevance from the point of diagnosis of any type or stage of cancer, providing access to holistic and supportive care alongside, for example, supporting people with decision-making around treatment choices and future care preferences [16]. In the context of SSA, palliative cancer care comprises a mix of generalist and/or specialist palliative care. Generalist (or universal) palliative care is care provided by any healthcare professional as an integral part of standard clinical practice to those affected by life-threatening diseases outside specialist palliative care. Specialist palliative care is care delivered by people with specialist training and expertise in palliative care. In SSA, both generalist and specialist palliative care are integrated into health facilities and home- or community-based programmes but are often delivered as a standalone programme [11]. A recent Lancet Oncology commission on cancer in SSA highlighted models of care that can be reproduced, adapted, and further developed across the region to reduce growing cancer crises [17]. Recommendations arising from the commission include telemedicine and mobile phone applications as strategies and innovations to prioritise palliative cancer care [17]. This is intended to improve the quality of and access to services, with recommendations for mobile-phone-based interventions, telehealth support and basic electronic medical records to be in use across services by 2025 [17]. Digital approaches may provide routes to addressing ongoing priority areas for palliative cancer care, including pain and symptom control (which are essential so that a holistic approach to care is possible) [18] and the need to optimise symptom management [19].

Recommendations for the development of digital approaches to support palliative cancer care in SSA build on increasing evidence of their potential to improve access to health services, be acceptable to patients and their caregivers, reduce costs and strengthen health systems [20,21]. Furthermore, the wider policies and infrastructures of SSA are supportive of digital health approaches. For example, across countries in SSA, there is an increasing number of national eHealth policies (e.g., [22,23]), greater mobile phone ownership, and better infrastructure (e.g., 70% coverage for 3G networks and 34% for 4G across SSA and recent 5G trials in Uganda and Nigeria [24]). There are also ongoing efforts to improve the affordability, content, services, and consumer readiness for digital technologies in the region [24]. In this opinion article, we bring together researchers, clinicians, technology developers and digital innovators to outline digital health approaches being developed for palliative cancer care in SSA. We focus specifically on remote symptom monitoring and outline three interventions from three countries, highlighting their models of delivery and intended outcomes. We draw on the experiences of implementation to guide further developments and evaluations of remote symptom monitoring for palliative cancer care in SSA. Through highlighting these current experiences and priority areas for future research and development, we hope to steer efforts to develop and optimise this approach for palliative cancer care in SSA.

## 2. Remote Symptom Monitoring for Palliative Cancer Care in Sub-Saharan Africa

Multiple technology applications have been highlighted as having a potential role in supporting cancer care in LMICs [1]. More recently, the COVID-19 pandemic has further increased the exploration of the use of technology for cancer care delivery, providing insights into and options for its implementation and utilization in LMICs [25,26]. Potential technological applications include point-of-care diagnostics (e.g., imaging tools such as portable ultrasound and treatment tools such as gasless cryotherapy), telemedicine (including telepathology and teleradiology), image analysis and pattern recognition for pathology and radiology, virtual reality in training, and digital applications to support the remote monitoring of patients through patient-reported outcomes [1]. In this manuscript, we focus on digital approaches for the remote monitoring of patients as a valuable approach to understanding the physical, psychological, and informational needs of people living with advanced cancer [27,28]. Such an approach can guide the development of locally appropriate approaches to care through the simultaneous characterization of the experiences, preferences, needs and outcomes of patients with cancer and their caregivers as well as the changes in these over time [29,30,31].

In the context of cancer care in high-resource settings, the evidence base underpinning remote symptom monitoring has been developing. For example, electronic patient-reported outcome approaches have demonstrated improved HRQoL and increased overall survival [32], alongside other benefits including improved communication between patients and clinicians [33] and more efficient symptom assessment [34]. Mechanisms through which remote symptom monitoring improves patient and caregiver outcomes are starting to be determined through research. This includes linkages between patient reporting, clinician actions and subsequent improvements in patient outcomes (e.g., earlier detection, improved symptom management, reduced symptom burden and reduced distress) [35]. However, to date, research depicting how remote symptom monitoring leads to changes in outcomes has been based on research evidence from high-resource settings [35]. In countries in SSA, there is evidence of the acceptability and feasibility of digital health approaches for palliative cancer care, including those that involve remote symptom monitoring [21]. However, challenges to cancer care delivery in SSA are multi-faceted and influence the delivery of screening, diagnosis, and treatment, such as the high cost of cancer medicines, high out-of-pocket expenses for cancer medicine incurred by patients and a lack of skilled training in treatment modalities including surgery [17]. Below, we present a conceptual map, adapted from remote symptom monitoring research in the US [35], to incorporate common challenges involved in the delivery of palliative cancer care in SSA [36]. This includes preliminary suggestions of causal links between remote symptom monitoring approaches and hypothesised pathways in which they may support the management of common issues experienced by patients with cancer in SSA.

**Figure 1:**
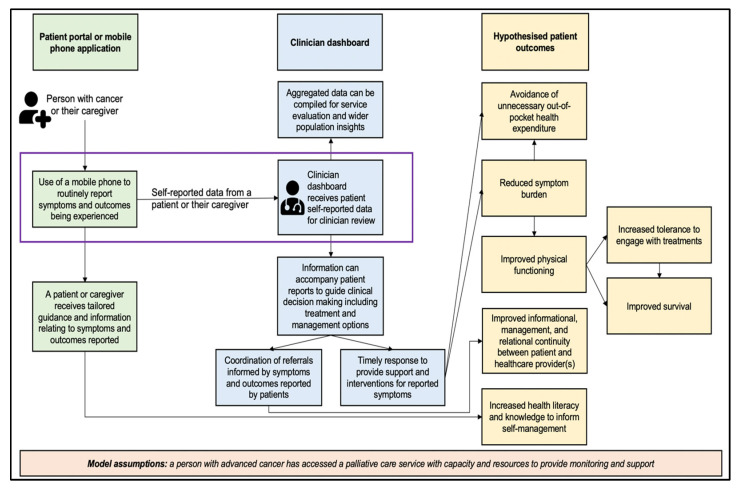
A schematic of hypothesised patient outcomes that may be derived from remote symptom monitoring for palliative cancer care in SSA. Informed by [35] and adapted to reflect common challenges relating to the provision and utilization of palliative care in Africa [36,37,38,39,40,41,42]. The purple rectangle indicates the focus of the three remote symptom monitoring approaches detailed in this manuscript (i.e., facilitated sharing of patient-reported outcomes data to a clinical team).

Multiple approaches and adaptations could be explored for remote symptom monitoring for palliative cancer care in SSA. In this manuscript, we report case studies from three pilot projects. Each of the case studies details an approach that facilitates communication between a person with cancer (or their caregiver) and a clinical team (reflected using a purple outline in Figure 1). Case studies reflect remote symptom monitoring used to facilitate links between patients and the following: (i) generalist palliative care providers in a cancer centre (Case Study 1), (ii) specialist palliative care providers alongside local health workers (Case Study 2), and (iii) generalist palliative care providers to guide referrals and coordination with other clinical settings that include specialist palliative care providers (Case Study 3). In all case studies, a person with cancer (or their caregiver) provides information via a mobile phone which is shared with a clinical team and presented via a dashboard. Figure 1 presents hypothesised pathways of how remote symptom monitoring and the sharing of information in this way may influence the management and outcomes of patients receiving palliative cancer care. Common issues experienced by people living with advanced cancer in SSA are reflected in the conceptual map. These include out-of-pocket expenditure for symptom management [37], a variable and mostly high symptom burden [38], decreased physical function [39], information needs relating to living with cancer [40], a need for improved continuity of care such as through support with navigating services [41] and low health literacy [42]. The case studies outlined below describe findings indicative of the feasibility and acceptability of this approach. Future research is required to explore and test hypothesised pathways and their impact on patient outcomes.

## 3. Case Studies

### 3.1. Case Study 1: Patient-Reported Outcome Side Effect (PROSE) Platform in Nigeria

Every year, more than 125,000 new cancer cases are recorded in Nigeria, with three-quarters of cancer cases diagnosed at a late and advanced stage [43]. Cancer therapies—radiotherapy, surgery, immunotherapy, and chemotherapy—although effective and increasingly available, are associated with acute and late side effects that significantly interrupt treatment, decrease adherence, lead to poor HRQoL and can at times be life-threatening [44,45].

Aligned with Figure 1, this approach to standardizing side effect reporting, documentation and capturing patients’ perspectives via a mobile phone application was established to support the timely identification of and response to patient side effects. This may lead to a reduced symptom burden, subsequently improving patients’ physical function and ability to maintain engagement with treatment, whether delivered with curative or palliative intent. Furthermore, information shared with patients about the management of their condition may increase the knowledge and capacity of patients to self-manage aspects of the side effects experienced during cancer therapies.

**Figure 2:**
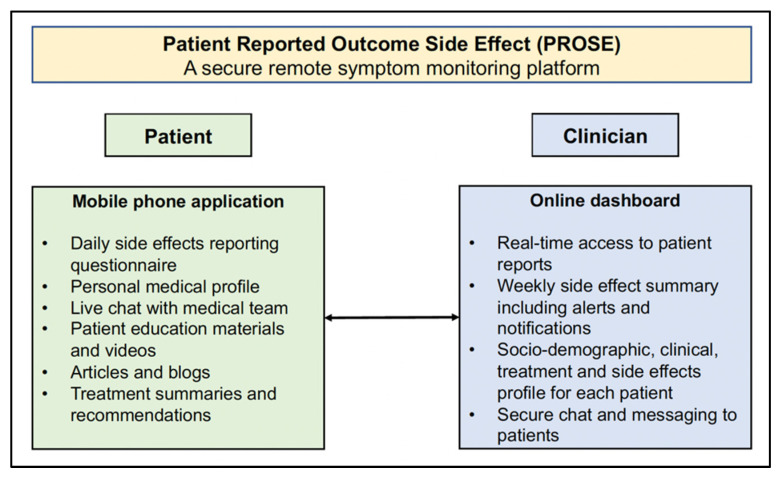
Overview of the PROSE intervention for side effect reporting during cancer treatment in Nigeria.

Researchers from the College of Medicine at the University of Lagos and Oncopadi Tech have developed an electronic patient-reported outcome tool that has been piloted at the NSIA-LUTH Cancer Centre, the largest comprehensive cancer centre in West Africa. Figure 2 displays the functionalities of the PROSE intervention for the patient and the clinician, including daily side effect reporting and education for patients, as well as real-time access to patient reports and side effect summaries for clinicians.

The intervention development commenced with focus groups and in-depth interviews with various stakeholders, including patients, informal caregivers and a radiation oncology team of nurses, oncologists, and therapy radiographers. Their insights and preferences in the use of technology were critical to developing the PROSE intervention.

To date, key lessons have included the need to understand patient and caregiver users of PROSE. Informal caregivers, in the context of intervention development, were defined as family members and friends who provide care to patients and play a key role during clinical and non-clinical hours. Recognizing the role of caregivers in study recruitment and their role in supporting reporting side effects on behalf of the patients to the PROSE app was vital in developing and implementing the PROSE tool. As patients experience debilitating treatment-related side effects, they rely on their caregivers to provide care and seek clinical advice and care to relieve them. Using the PROSE platform allowed both patients and caregivers to be involved and active in the patients’ care. The inclusion of caregivers in the tool’s utilization may have contributed to patient engagement during PROSE pilot testing, with a 62% utilisation rate. The utilisation rate was defined as >50% of daily reports completed during a 6-week study period by patients following the submission of an initial report. The high PROSE utilization rate provides reassurance that the remote symptom monitoring approach could be feasible with promising levels of engagement. Further development work is underway to understand the barriers and characteristics of those not taking up or maintaining the use of PROSE, alongside the exploration of optimal approaches to relay data to patients in real-time, in a valuable and meaningful way.

### 3.2. Case Study 2: m-Palliative Care Link for Palliative Care Coordination among Patients with Cancer in Tanzania

In Tanzania, mobile phone ownership continues to increase alongside mobile broadband coverage (currently estimated at 81% of the population, up from 1% in 2006) [46]. Coupled with a projected further increase in smartphone ownership, mobile health [3] promises to increase access to palliative care specialists, resulting in improved symptom management among patients with cancer in Tanzania, this study setting, and other low-resource settings [47]. We worked closely with various stakeholders in Tanzania including physicians and nurses specializing in palliative care, patients, informal caregivers, and local health workers (LHWs). A human-centred design (HCD) framework was followed to co-design, develop, and validate the usability of the mobile-Palliative Care Link (mPCL) prototype. mPCL, a linked web-based platform and mobile phone application, focused on assisting Tanzanian patients with cancer who have a poor prognosis by providing symptom assessment and management. This technological approach addresses the challenge of the limited number of palliative care specialists and community-based LHWs [48], thus expanding the specialist input in the management of people living with cancer. mPCL aims to facilitate the timely identification and management of symptoms to address and alleviate any symptom burden, similar to elements outlined in the conceptual map in Figure 1. 

**Figure 3:**
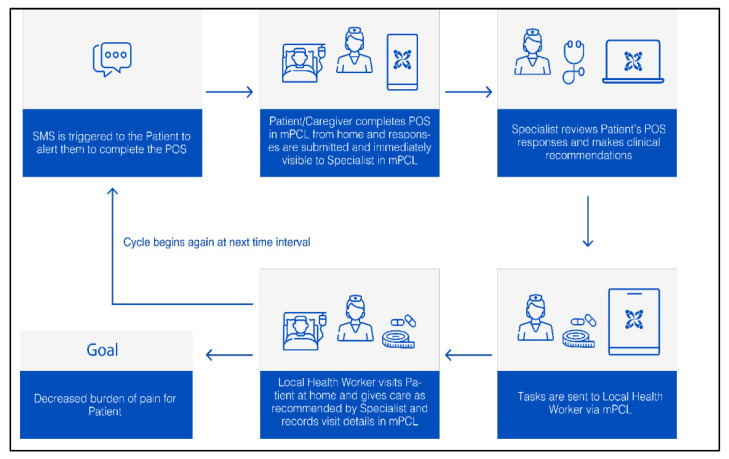
Design of the mPCL app focusing on the patient or informal caregiver and care team communication and care coordination. mPCL: mobile-Palliative Care Link; POS: Palliative Care Outcome Scale.

mPCL is depicted in Figure 3, central to which is the validated African Palliative Care Outcome Scale (POS) [49]. This is a 10-item multidimensional tool to assess the physical and psychological symptoms, spiritual, practical, and emotional concerns, and psychosocial needs of patients and their families. The tool was adapted for the automated, twice-weekly collection of quality-of-life-focused patient and caregiver responses and timely reviews, reactions and tracking by specialists and LHWs. mPCL is expressly designed to facilitate coordinated care via customized interfaces supporting core users—patients or caregivers, LHWs and members of the palliative care team—and their respective roles. Usability testing revealed general mPCL acceptance, and early pilot testing showed usability and feasibility in supporting outpatient palliative care for Tanzanian patients in the setting of a single urban cancer institute [50,51].

Future work is needed to demonstrate the effectiveness and sustainability of mPCL to support the symptom control needs of a broader population of Tanzanian patients with cancer, particularly in harder-to-reach areas. Additional questions related to this work include the generalizability of mPCL to settings with less access to symptom-control medications and other support resources. As mobile technologies continue to grow and evolve in low-resource settings such as Tanzania, the field of cancer medicine can greatly benefit from an understanding of how to build patient-centric tools optimized for remote symptom monitoring and tracking as well as effective and efficient care coordination. Rigorous studies of the use of these tools in practice are critical to understanding how they can be widely adopted and scaled.

### 3.3. Case Study 3: African Palliative Care Association (APCA) Mobile Phone Application and Clinician Dashboard for Supporting Continuity of Care for People Living with Cancer in Refugee Settlements in Uganda

Work undertaken by the African Palliative Care Association in partnership with the University of Leeds highlighted key requirements to consider in the design and evaluation of digital health approaches for palliative care in SSA [52]. These requirements were used to develop a feasible approach to supporting continuity of care for patients with palliative care needs in refugee settlements during the COVID-19 pandemic. The setting of refugee settlements was chosen due to palliative care not currently being an integral part of humanitarian and emergency responses. This work aligns with a wider need to develop the integration of palliative care provision into existing health and social care systems and the broader humanitarian health response [53].

**Figure 4:**
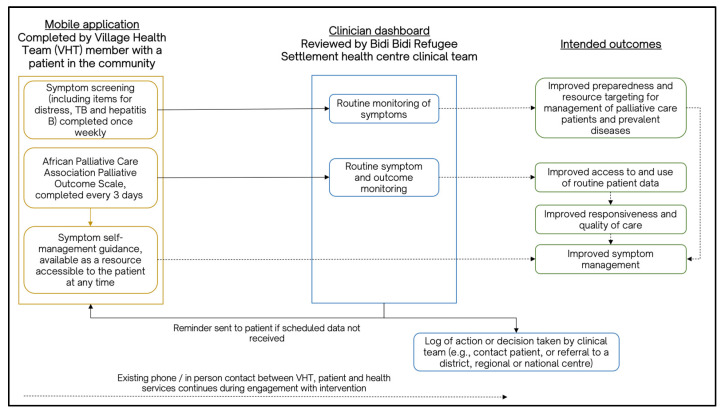
Schematic of the African Palliative Care Association mobile phone application and clinician dashboard.

The digital health intervention includes two components: a mobile application that is completed by a health worker from the settlement clinical team alongside the patient; and a web-based dashboard for the clinical team that displays the data gathered from the mobile application (as shown in Figure 4). Together, using the mobile application, a health worker and patient complete a symptom screening questionnaire and a multidimensional outcome measure designed for palliative care in Africa [49]. The data entered into the mobile application are then shared on a clinician’s dashboard, enabling a settlement clinical team to use reports for the routine monitoring of patients, determine any changes or problematic symptoms, and devise an appropriate response plan. This may involve coordinating with external palliative care specialists located beyond the settlements to ensure comprehensive cancer care. Aligned with Figure 1, this approach seeks to support the timely identification and management of symptoms and issues, as well as inform the coordination of and referrals to additional services and clinical sites when needed. This may potentially influence reductions in any symptom burden experienced by patients, as well as increase coordination and continuity of care.

To date, two pilot studies have been conducted with patients living with advanced cancer (n = 35) in Bidi Bidi Refugee Settlement and Adjumani Refugee Settlement in Uganda. The studies have demonstrated the feasibility of implementing the intervention in the context of the settlements. Semi-structured interviews with patients highlighted the high value attributed to the additional time spent with health workers resulting from the intervention, with scope for structured discussions around any problems or concerns that they have experienced. Semi-structured interviews with health workers highlighted the benefits of patient self-management information included in the mobile application, enabling them to work through resources with patients and supporting the implementation of their guidance. Clinical teams valued being able to review patient progress remotely, whilst also being able to track health worker interactions with patients and their carers. Furthermore, the clinical teams valued the data captured on tuberculosis and hepatitis B; the collection of these items was requested by clinical teams during the development phase of the intervention. The next steps for development include (i) the further evaluation of the intervention and its impact on patient care and outcomes; (ii) pilot testing of the intervention at additional settlements to understand the processes around its scale-up and adoption beyond existing sites; and (iii) revising content on COVID-19 and developing resources capturing common symptoms and concerns, including distress, using validated tools, such as the distress thermometer [54].

## 4. Developing the Evidence Base to Inform Future Research on Remote Symptom Monitoring for Cancer Care in SSA

There is a commonality across the different case studies presented above: rich patient-level data are captured to inform the management of people with advanced cancer. When information is shared with clinicians, this provides opportunities to increase the timeliness and personalisation of care. In addition, all interventions encourage engagement with information to enhance the capability of people with cancer to self-manage aspects of their condition, often with support from informal caregivers. To guide the development of remote symptom monitoring approaches for cancer care in SSA, below we highlight key target areas for future research derived from our collective experiences of developing and implementing remote symptom monitoring approaches.

### 4.1. Develop New Care Models and Accompanying Clinical Workflows

In the context of palliative cancer care in SSA, the three remote symptom monitoring approaches presented above are generating evidence to support their acceptability and feasibility. Beyond their development, we will need to understand how models of care delivery can be adapted to accommodate the integration of remote symptom monitoring. This is particularly important in SSA, where digital health interventions are generally at an early stage of development [55]. Remote monitoring approaches (or patient-centric digital solutions in general) would be considered in later stages of maturity for electronic medical record technologies. For example, remote monitoring is aligned with the latest stages (i.e., 6 and 7) of the Electronic Medical Record Adoption Model developed by the Healthcare Information and Management Systems Society [56]. Furthermore, there are few interventions reported across the literature that focus on gathering individual-level clinical outcome data from patients for oncology care in Africa [57]. This provides opportunities for advancing research to explore digital approaches to patient-level data collection (including symptom reporting) across both adult and paediatric palliative cancer care.

When considering the incorporation of remote symptom monitoring into clinical workflows, it may be necessary to account for the existing inertia towards adopting technology and the lack of funding that hinders its development [58]. In the context of high-resource settings in which the evidence base underpinning remote symptom monitoring is more established, there remains a need to develop strategies for the routine assessment and implementation of patient-reported outcomes in clinical practice [59]. Incorporating remote symptom monitoring into routine clinical care, at scale, across all settings relevant to palliative care delivery in SSA can be expected to require considerable staff engagement, modifications to the healthcare delivery system and sustained investments, given the complex nature of the approach [35].

### 4.2. Determine the Economics of Digital Health Approaches for Palliative Cancer Care in SSA

As part of developing new care models, there is a need to adapt feasible approaches to cost analyses for palliative care in SSA [60] to explore the economic framing of the benefits derived from remote symptom monitoring in low-resource settings. This can support the development of hypothesised pathways relating to unnecessary health expenditure, as detailed in the conceptual map in Figure 1. In SSA, people with advanced cancer often have limited or no social protection systems to provide a safety net from financial toxicity. This often imposes the need to pursue coping strategies that are not sustainable and increase the risk of bankruptcy [61]. Our understanding of the economic impact of remote monitoring across the levels of patients, caregivers, households, communities, health services and wider health systems is currently lacking but is an important component for guiding the development of realistic, long-term funding models. Traditionally, healthcare quality has been assessed in terms of mortality or similar objective measures. As many African countries are implementing health insurance schemes and value-based care reimbursement, patient-reported outcomes (e.g., remote self-monitoring of symptoms) will become increasingly relevant as metrics to determine performance and reimbursement, which consequently could motivate or boost improvements in care for people living with advanced cancer [62,63]. A further economic element to consider is the cost of the devices themselves, which are critical to enabling digital health interventions. Research is required to explore which technologies may be appropriate in the absence of smartphones, such as basic mobile phones, and whether equitable outcomes can be achieved for patients irrespective of the type of device used as part of a digital health intervention.

### 4.3. Determine Capacity and Capability to Implement and Sustain Platforms to Support Remote Monitoring in Palliative Cancer Care

The interventions outlined above have, to date, been supported through research funding to enable their development and testing. Digital health innovation and development needs to consider coordination, sustainability, regulation, and interoperability often at the policymaker level to avoid fragmented and short-term digital health systems [64]. In the context of low-resource settings, best practices around digital health development includes the ability to integrate into an existing district and national digital health platforms, alongside having committed, long-term funding that facilitates digital innovations to iterate, evolve and be embedded into existing systems and practices [65]. The remote symptom monitoring approaches described above are standalone interventions, developed independently of existing electronic health records and district and national health management information systems, which tend to be weak across SSA (i.e., having limited breadth and quality of data relating to palliative cancer care, including the characterisation of the disease and its burden, such as the number of cases and impact on patient outcomes) [66]. Future developments of remote symptom monitoring will need to consider longer-term plans for integration, storage, and data sharing across existing platforms.

The integration of aggregated data from remote symptom monitoring approaches into information systems used by both health professionals and policymakers may help to address existing challenges in accessing quality evidence to inform planning, decision making and resource allocation for services including cancer care in SSA [67,68]. However, this may require supplementary education programs to train individuals and organisations to develop, implement, maintain, and contribute to the development of remote symptom monitoring approaches, alongside the need for technical support and resources to facilitate and support all stages of platform development and rollout [69]. Additionally, at the health professional level, there is a need to design systems that capture and report information for which there is the capacity to act. Capacity may be influenced by multiple factors (e.g., human resource limitations and medication supply issues) in the context of overstretched health systems. Equipoise around data collection needs to be sought. Questions to explore might include: How often should data be captured? Which data are crucial to capture and how can systems prioritise alerts for the outcomes that are critical? What frequency and magnitude of alerts from remote monitoring systems are manageable and workable? And who are the preferred recipients of alerts and information—are these the people with the capacity to review and respond?

### 4.4. Consider the Potential of New Technologies and Their Relevance and Utility for Palliative Cancer Care in SSA

The remote symptom monitoring approaches outlined in this article currently use a mobile phone interface, with data shared with a web-based clinician interface. As new technologies arise, it may be useful to explore their relevance in supporting patient, clinical or service-level needs that can be feasibly supported within palliative cancer care delivery in SSA. For example, wearable trackers are emerging to enhance remote monitoring and the passive capture of objective data that can augment information presented and available to inform clinician decision-making [70]. This might be particularly helpful in the terminal palliative phase. Including a wearable device as part of a digital health intervention has supported significant improvements in outcomes for people with advanced cancer, including their levels of physical activity, HRQoL and fatigue [71]. Wearable devices have been demonstrated to be well accepted and capable of gathering continuous measurements in a study in Burkina Faso, but their potential for palliative cancer care has yet to be explored [72]. Wearables, however, often require internet and smartphones which account for less than half of all mobile phone connections in Africa [73]; basic mobile phones continue to dominate. A further set of promising new technologies that face limitations in the African context is conversational artificial intelligence (AI) chatbots. These can be used for the collection of patient-reported outcomes (including remote symptom monitoring) as well as providing patient education, counselling, and psychological support [74]. However, in addition to the limited access to the internet and smartphones mentioned above, conversational AI may have limited support for African languages, including the ability to understand and respond appropriately to diverse cultural nuances and dialects specific to each region. Therefore, innovations that harness widely available infrastructure and functionality (e.g., basic telephone calls, interactive voice response (IVR) system, or Unstructured Supplementary Service Data (USSD)—an approach already widely used for mobile phone-based financial transactions) remain attractive and equitable. Moreover, a few studies have demonstrated the application of these basic mobile-phone-based tools to support cancer patients and their caregivers [75] and people living with other advanced diseases [76], alongside being reported as acceptable amongst clinicians, patients, and caregivers.

### 4.5. Explore the Unknown Feasibility of Emerging Experimental Methodologies for Digital Health in Oncology Care in SSA

Feasibility and acceptability testing has been completed on the three examples of remote monitoring outlined above. However, there remains a need to consider optimal approaches to evaluating their use in palliative cancer care in SSA. One aspect is the outcomes used during evaluations. Across oncology care research in SSA, digital health evaluations typically comprise isolated pilots focused on structure and process measures (e.g., acceptability and ease of use) [57], similar to the pilot work outlined in the case studies above. The impact of remote symptom monitoring and other digital health approaches on clinical oncology outcomes, such as cancer incidence or mortality, has not been investigated [57]. Furthermore, longer-term outcomes that include the scale-up and sustainability of remote monitoring approaches should be considered [77]. Implementation science approaches will be crucial in supporting this, moving beyond evaluating the efficacy of remote symptom monitoring and digital health approaches. This will enable a focus on understanding their effectiveness, including how to ensure that these innovations have sufficient reach, adoption, fidelity, sustainability and economic utility [78,79].

Remote symptom monitoring, alongside digital health approaches, is typically a complex intervention with multiple components (e.g., symptom reporting, self-management guidance and support). Methodological developments are underway, allowing for a more systematic and refined investigation into such interventions. Experimental approaches, such as factorial designs, sequential multiple assignment randomized trials and micro-randomized trials [80], offer valuable opportunities to evaluate multi-component interventions and provide scope to explore these novel digital interventions for oncology care in SSA. Alongside being assessed, remote symptom monitoring approaches may also provide a useful tool for use within trials. Through gathering patient-reported outcomes data, they could be useful tools for clinical trials across SSA to support context-specific high-quality evidence on which to base treatment decisions, clinical guidelines, and resource allocation [1]. 

### 4.6. Monitor Inequalities in Access to and Benefits from Remote Monitoring Approaches

While findings from the early testing of the three remote symptom monitoring approaches outlined above are encouraging, consideration needs to be provided to their further development to ensure inequalities are not further exacerbated and enforced through such digital approaches. In SSA, a diagnosis of cancer can subject vulnerable households to catastrophic health-related costs [81]. In this context, the introduction, use and potential impact of digital health approaches need to be considered. Recent lessons from India may have relevance to SSA, where socio-economic characteristics such as age, literacy and sociocultural norms remain major barriers to the equitable use of digital technologies [82]. Furthermore, cost alone remains challenging, with people unable to afford to own a phone, subscribe to a phone service, or recharge a phone regularly. There is a need for research to better understand equitable approaches to the delivery of remote symptom monitoring for palliative cancer care in SSA. 

All stages of cancer are associated with stigma among patients, so innovations should be designed to reduce stigma and enhance support through, for example, telehealth workflows or features that facilitate caregivers’ participation in the patient’s care. There is a need to ensure approaches are context-specific and reflect the needs of local populations—from the languages, content, and cultural references within the content, to who collects and reports data. Furthermore, the ongoing monitoring of access and utilisation, as well as the differential impact across different subgroups may require continued monitoring during testing and a wider rollout of symptom monitoring approaches. The identification of potential challenges during the development and before the implementation of remote symptom monitoring approaches can be supported through the involvement of intended users in their development; user involvement is feasible and forms a critical element in intervention development [21,83].

### 4.7. Measure Health System Benefit(s) and Integration

Remote monitoring approaches may be an approach to address the dearth of data on the experiences, preferences, needs and outcomes of patients with advanced cancer and their caregivers in SSA, including how these change over time [29,30,31]. These data are crucial to guiding the development of locally appropriate approaches to delivering palliative cancer care in SSA. Remote symptom monitoring approaches could provide a means of developing methodologically rigorous processes for gathering data to develop the evidence base specific to palliative cancer care in SSA. This could include generating accurate and real-time data on symptoms and disease burden for patients with advanced cancer, alongside strengthening underpinning data to inform clinical trials [84] and routine care delivery [85]. Furthermore, there may be value in aggregate and population-level summaries of data gathered through remote symptom monitoring approaches. For example, this could provide adjunct data that are useful for service planners, commissioners, and policymakers to inform service evaluation and planning. The integration of remote symptom monitoring data into emerging electronic record systems or aggregated data into health information systems across SSA may provide a means of strengthening cancer intelligence for the region.

### 4.8. Build and Foster Partnerships

We present work from a growing community of practice exploring the role of remote monitoring for palliative cancer care in SSA. Partnerships and collaboration are key to supporting the further development of our work. Coordinating activities and learning provides opportunities for knowledge sharing and the potential for increasing the scale and impact of our work. Existing networks provide a forum for sharing our findings and for engaging other researchers, clinicians, technology developers and others interested in remote symptom monitoring. For SSA, organisations such as the African Organisation for Research and Training in Cancer (AORTIC) provide routes to having an impact through the dissemination of findings and training expertise to guide how digital health literacy and capabilities can be developed for cancer care broadly in SSA. International organisations such as the Multinational Association of Supportive Care in Cancer (MASCC) enable the fostering of collaboration and sharing of best practices on supportive care in cancer that may have relevance to SSA. Such international organisations can pay more attention to making the products (e.g., guidelines, patient information) applicable and available to low-resource settings and match them with locally available resources. The development and validation of remote symptom monitoring tools, translation of tools to local languages and contexts, and adaptation of tools for different platforms (e.g., audio questionnaires to be distributed via IVR for patients with limited reading literacy) will benefit from the collaboration and distribution among (inter)national researchers and clinicians. Working with initiatives in this domain (e.g., the International Consortium for Health Outcomes Measurement and the Patient Reported Outcomes Measurement Information System) could expedite the development and evaluation of remote monitoring for patients accessing palliative cancer care in SSA. 

## 5. Conclusions

This article brings together a community of researchers and clinicians developing digital health and remote symptom monitoring approaches for palliative cancer care in SSA. We present three case studies of remote symptom monitoring interventions piloted across the region to highlight how they could enhance cancer care delivery in SSA. The receipt and use of timely data afforded by remote symptom monitoring—direct from the patient or their caregiver—are necessary to inform efforts to improve the quality of services and measure impact [86,87]. Useful, relevant, and reliable data are needed to characterise the current symptom and disease burden experienced by people living with cancer in SSA [85] and understand the extent to which service delivery is aligned with the needs of the population being served [30]. Furthermore, access to timely, reliable, and practical health information is a prerequisite for delivering universal health coverage [88]. However, multiple factors require further consideration and research to guide the development of remote symptom monitoring for palliative cancer care in SSA. These include the augmentation of care delivery models, how to sustain digital platforms as part of routine care, the construction of new economic models for digital health approaches, monitoring inequalities that may arise through their use, and further developing and fostering partnerships to grow the community of practice around remote symptom monitoring for palliative cancer care in SSA.

## Data Availability

No new data were created or analyzed in this study. Data are contained within the article.
